# Identification of a RNA-Seq based prognostic signature with five lncRNAs for lung squamous cell carcinoma

**DOI:** 10.18632/oncotarget.17098

**Published:** 2017-04-13

**Authors:** Rui-Xue Tang, Wen-Jie Chen, Rong-Quan He, Jiang-Hui Zeng, Liang Liang, Shi-Kang Li, Jie Ma, Dian-Zhong Luo, Gang Chen

**Affiliations:** ^1^ Department of Pathology, First Affiliated Hospital of Guangxi Medical University, Nanning, Guangxi Zhuang Autonomous Region, P. R. China; ^2^ Department of Thoracic and Cardiovascular Diseases, First Affiliated Hospital of Guangxi Medical University, Nanning, Guangxi Zhuang Autonomous Region, P. R. China; ^3^ Department of Medical Oncology, First Affiliated Hospital of Guangxi Medical University, Nanning, Guangxi Zhuang Autonomous Region, P. R. China; ^4^ Department of General Surgery, First Affiliated Hospital of Guangxi Medical University (West Branch), Nanning, Guangxi Zhuang Autonomous Region, P. R. China

**Keywords:** lncRNAs, LUSC, biomarker, prognosis index, overall survival

## Abstract

Long non-coding RNAs (lncRNAs) expression profile signature for survival assessment in lung squamous cell carcinoma (LUSC) are largely inconsistent due to distinct detecting approaches and small sample size. Systematic and integrative investigation of RNA-Seq based data from The Cancer Genome Atlas (TCGA) herein was performed to determine candidate lncRNAs for prognosis evaluation of LUSC. A total of 60483 genes, including 7589 lncRNAs were assessed in a cohort including 478 LUSC cases with follow-up data. Firstly, 4225 differentially expressed lncRNAs were obtained via R packages. Next, univariate and multivariate Cox proportional hazards regression revealed that 41 lncRNAs were closely related to the survival of LUSC. Finally, lncRNA based prognosis index (PI) could predict overall survival of LUSC with high accuracy (AUC = 0.652, CI: 0.598, 0.705), PI = exp_CYP4F26P_*β_CYP4F26P_+exp_RP11-108M12.3_*β_RP11-108M12.3_+exp_RP11-38M8.1_*β_RP11-38M8.1_+exp_RP11-54H7.4_*β_RP11-54H7.4_+exp_ZNF503-AS1_*β_ZNF503-AS1_. Furthermore, it was confirmed that the five-lncRNA signature could act as an independent prognostic indicator for LUSC (HR = 2.068, *p* < 0.001 with univariate analysis, HR = 1.928, *p* = 0.038 with multivariate). Besides, we constructed a weighted gene co-expression network analysis (WGCNA) of key lncRNA RP11-54H7.4 according to the *p*-value of related genes’ weight. This study provides a RNA-Seq based prognostic signature with five lncRNAs for further clinical application to LUSC patients.

## INTRODUCTION

Lung cancer remains one of the most common causes of cancer-related deaths all over the world, with over 25% of all cancer-related deaths in both male and female [1–6]. Lung cancer includes two main histological categories: small cell lung cancer (SCLC) and non-small cell lung cancer (NSCLC) [7–4]. And nearly 80% of lung cancers are NSCLCs, which consists of different subtypes: lung adenocarcinoma (LUAD), lung squamous cell carcinoma (LUSC) and large cell lung carcinoma (LCLC). These different subtypes of lung cancer are related to distinct molecular profiling and are believed to originate from distinct cells [15–18]. Recent developments in molecular pathology detection and targeted therapies have markedly improved the overall survival (OS) of patients with LUAD. However, there are no specific biomarkers or comparable effective targeted molecular therapies have been available to screen and treat LUSC patients [19–21]. Thus, the survival rates of LUSC remain relatively low. In that regard, there is an urgent requirement for effective prognostic biomarkers and treatment options based on the current genomic approaches for LUSC.

Presently, the innovation of a particularly large number of non-coding RNAs conceptually transformed the field of cancer research, including long noncoding RNAs (lncRNAs). LncRNAs are non-protein coding transcripts of ≥ 200 nt, which are commonly distributed in the genome and can modulate gene expression [22–26]. Recent increasing evidence has revealed that lncRNA expression profiles are dysregulated in various cancers [27–31]. The aberrant lncRNA expression profile has been reported to correlate to the development and survival in patients with different kinds of cancers, including LUSC, which reveals the potential of lncRNAs as prognostic cancer biomarkers [32–36]. However, most of the previous studies focused on a single lncRNA based on small sample size [37–40].

We performed the current study using the LUSC dataset retrieved from The Cancer Genome Atlas (TCGA, http://cancergenome.nih.gov/) to construct a panel of lncRNA signature and obtain a specific prognosis index (PI), which could predict the prognosis in LUSC. These findings could also provide new insight into the molecular mechanisms based on lncRNAs for LUSC.

## RESULTS

### Differentially expressed lncRNAs in LUSC

**Figure 1:**
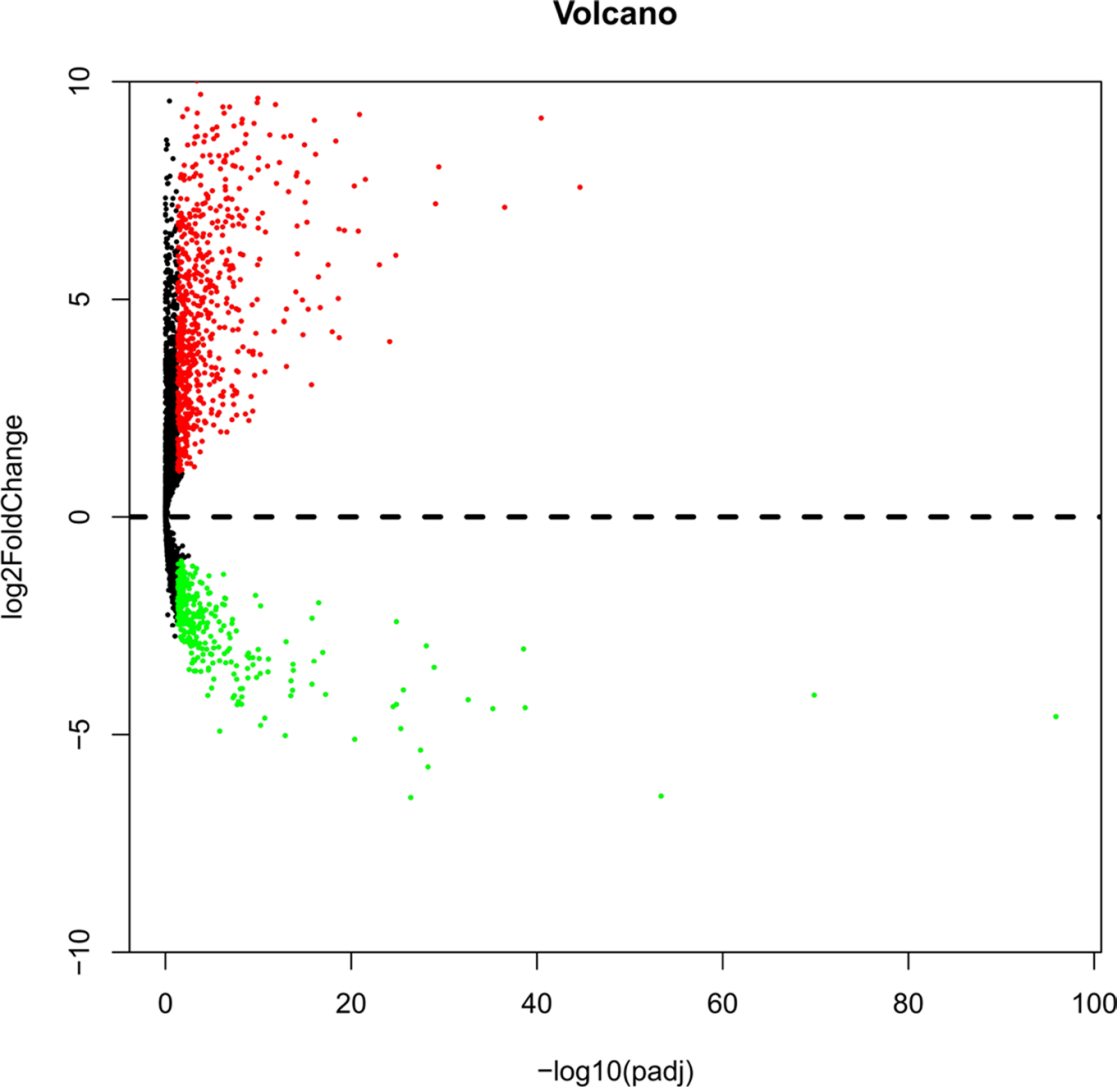
Volcano plot of the differentially expressed lncRNAs between LUSC and para-carcinoma tissues Red indicates high expression and green indicates low expression. Black shows the lncRNA expression with both the log2FC < 1 and adjusted value < 0.05. The X axis represents an adjusted *P* value and the Y axis represents a log2FC. Differentially lncRNAs were calculated by DESeqR 2809 high expressed lncRNAs, and 1416 low expressed. This volcano plot was conducted by the ggplot2 package of R language.

Among the expression data of 60,483 mRNAs, 7589 lncRNA expression was extracted and calculated with R language (EdegR and DESeq). Differentially expressed lncRNAs (*n* = 4225, Figure 1) were achieved, which were sent for further investigation on prognostic value.

### Prognostic evaluation of the differentially expressed lncRNAs and clinicopathological parameters

After we excluded the cases with insufficient survival data, we finally obtained 478 cases for the prognostic evaluation. If the expression data had 10% absence for an lncRNA, this lncRNA was also omitted from our prognosis evaluation. The univariate Cox proportional hazards regression method revealed that a total of 41 lncRNAs gained prognostic value for LUSC. Subsequently, multivariate Cox proportional hazards regression analysis was applied to verify the aforementioned results, and CYP4F26P, RP11-108M12.3, RP11-38M8.1, RP11-54H7.4 and ZNF503-AS1 were proved to be independent prognostic indicators for LUSC and their individual prognostic values were shown in Table 1. Next the prognosis index (PI) for predicting OS was calculated with the formula based on the five lncRNAs above:

**Table 1:** The detailed information of five prognostic lncRNAs significantly associated with overall survival in LUSC

Gene name	Ensemble ID	Chromosome	Estimate	SthErr	ChiSq	*P*-value
CYP4F26P	ENSG00000226562	Chromosome 9: 33,580,695–33,605,293	−0.00342	0.001264	7.305626	0.006873906
RP11-108M12.3	ENSG00000258592	Chromosome 14: 57,066,260–57,112,660	−0.01706	0.004641	13.5081	0.000237536
RP11-38M8.1	ENSG00000273297	Chromosome 7: 134,416,290–134,432,453	0.001686	0.000785	4.610329	0.031779934
RP11-54H7.4	ENSG00000275216	Chromosome 13: 109,269,634–109,273,838	3.2E-05	1.36E-05	5.522873	0.018769411
ZNF503-AS1	ENSG00000226051	Chromosome 10: 75,269,819–75,373,500	0.000816	0.000352	5.379276	0.020377331

PI = exp_CYP4F26P_*β_CYP4F26P_+exp_RP11-108M12.3_*β_RP11-108M12.3_+exp_RP11-38M8.1_*β_RP11-38M8.1_+exp_RP11-54H7.4_*β_RP11-54H7.4_+exp_ZNF503-AS1_*β_ZNF503-AS1_.

The “β” value is the estimated regression coefficient of lncRNA derived from the multivariate Cox stepwise regression analysis and “exp” indicates the expression profiles of lncRNA.

**Figure 2:**
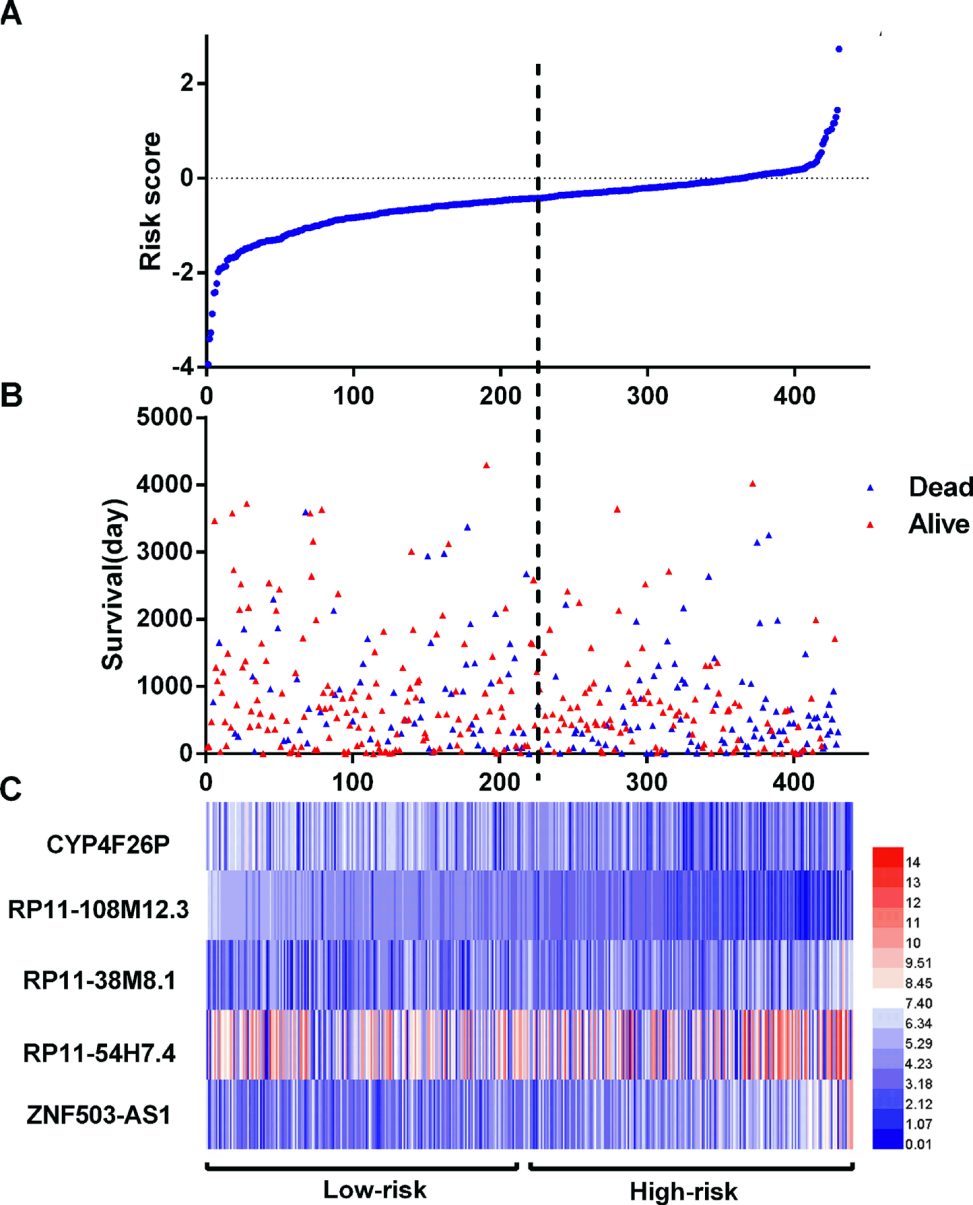
LncRNA risk score analysis of LUSC patients (**A**) The low and high score group for the lncRNA signature in LUSC patients; (**B**) The survival status and duration of LUSC cases; (**C**) Heatmap of the five key lncRNAs expression in LUSC. The color from blue to red shows a trend from low expression to high expression.

**Figure 3:**
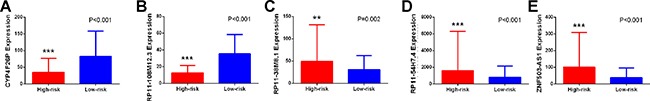
Different expression of the five key lncRNAs between high risk group and low risk group (**A**) CYP4F26P; (**B**) RP11-108M12.3; (**C**) RP11-38M8.1; (**D**) RP11-54H7.4; (**E**) ZNF503-AS1 *P < 0.05, **P < 0.01, ***P < 0.001, ****P < 0.000.

**Figure 4:**
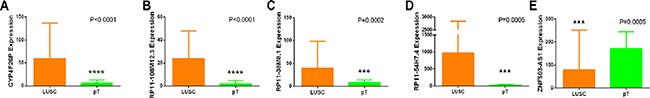
Different expression of the five key lncRNAs between LUSC and para-noncancerous lung tissues based on TCGA data (**A**) CYP4F26P; (**B**) RP11-108M12.3; (**C**) RP11-38M8.1; (**D**) RP11-54H7.4; (**E**) ZNF503-AS1. pT: para-noncancerous tissues. *P < 0.05, **P < 0.01, ***P < 0.001, ****P < 0.0001.

**Figure 5:**
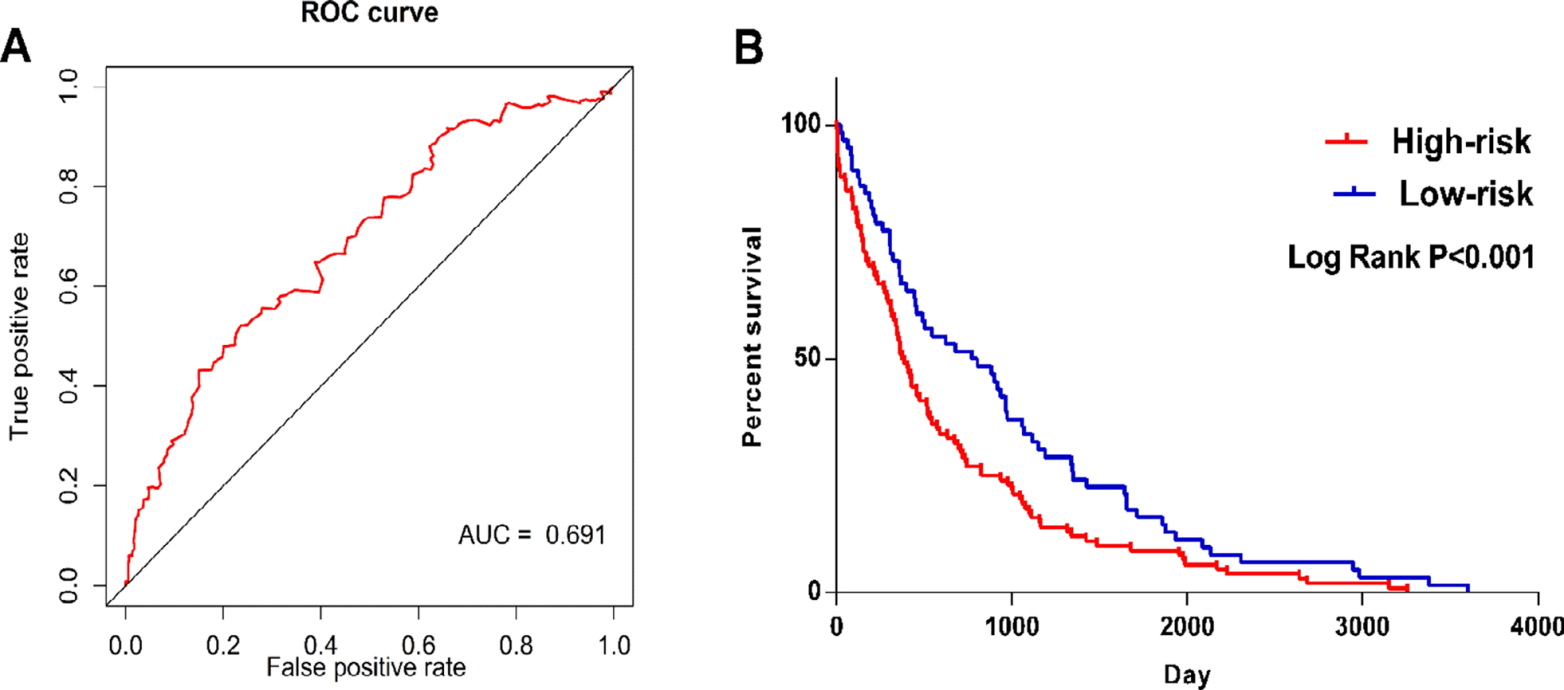
ROC and Kaplan–Meier curves for the five lncRNAs signature in TCGA LUSC cohort (**A**) Time-dependent ROC curves analysis for survival prediction by the five-lncRNA signature. (**B**) Kaplan-Meier survival curves showing overall survival outcomes according to relative high-risk and low-risk patients.

The LUSC patients were divided into two groups of low-risk and high-risk according to the median point of the prognostic risk score (Figure 2). We also assessed the difference of the expression levels in these five lncRNAs between high- and low-risk groups. Remarkably lower expression was noted for CYP4F26P and RP11-108M12.3 in high-risk groups, while higher expression was observed for RP11-38M8.1, RP11-54H7.4 and ZNF503-AS1 in high-risk groups (Figure 3). Original expression of these five lncRNAs between LUSC and non-cancerous lung tissues were also evaluated. Remarkably higher expression was noted for CYP4F26P, RP11-108M12.3, RP11-38M8.1, RP11-54H7.4, while predominantly lower expression was observed for ZNF503-AS1 in LUSC (Figure 4). The risk score could helpfully predict 5-year survival of LUSC patients, as the AUC of survival ROC reached 0.691 (Figure 5A). Furthermore, K-M curves indicated that the median survival time of patients of high-risk group was 30.92 months, far shorter than that of low-risk group 66.67 months, *p* < 0.001, Figure 5B). Additionally, the HR of the PI generated by univariate Cox proportional hazards regression method was 2.068 (CI: 1.503, 2.847, *p* < 0.001) and multivariate Cox proportional hazards regression analysis revealed a consistent HR of 1.928 (CI: 1.037, 3.583, *p* = 0.038), which confirmed that the PI of five lncRNAs could function as an independent indicator for the survival of LUSC patients.

**Figure 6:**
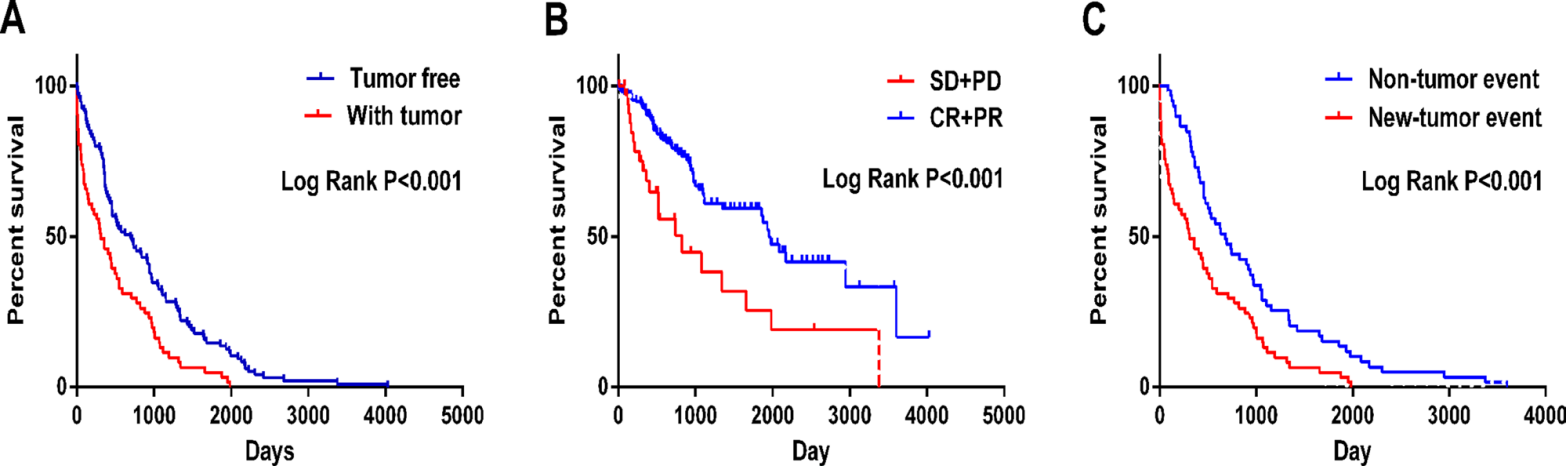
Kaplan-Meier survival curves in subgroup analyses according to different clinical factors (**A**) Cancer status (**B**) New tumor event after initial treatment (**C**) Primary therapy outcome success (SD: stable disease; PD: progressive disease; CR: complete response and PR: partial response).

**Figure 7:**
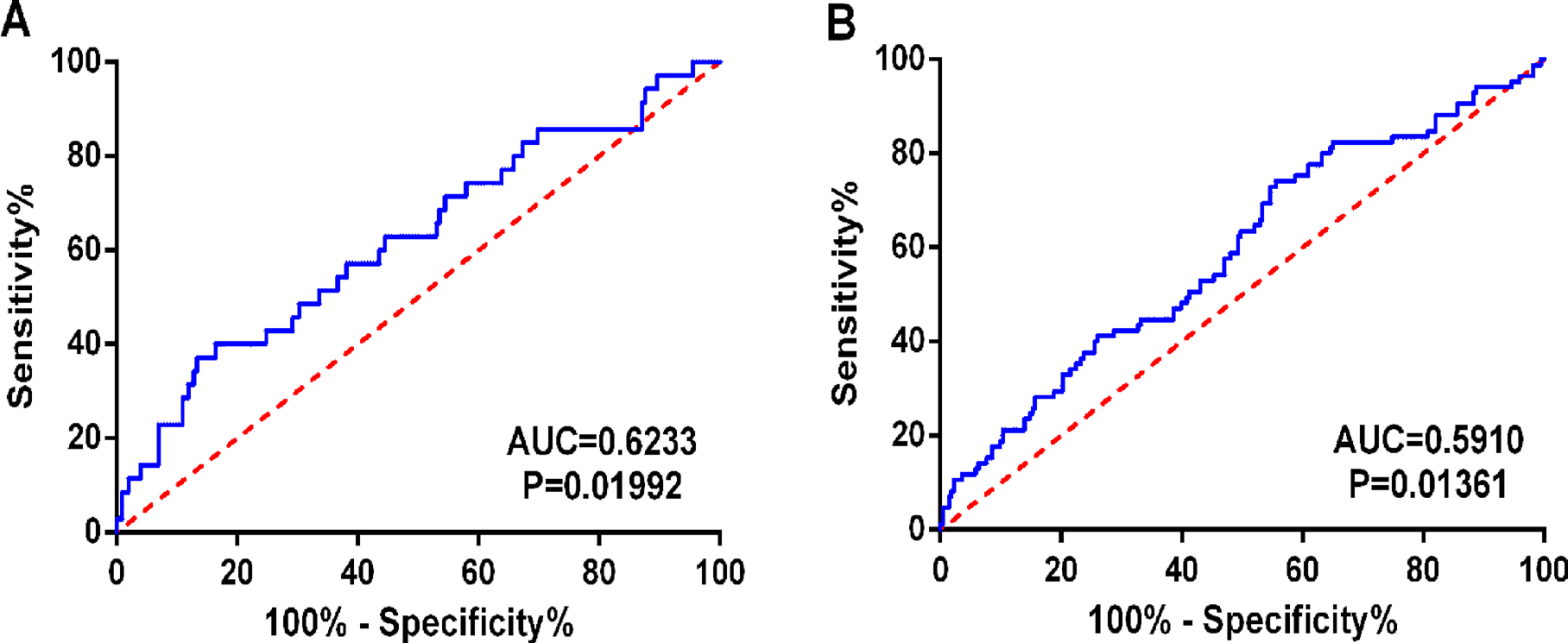
Predictive value of the risk scores for clinical features by ROC curves (**A**) New tumor event (AUC = 0.6233, *P* = 0.01992); (**B**) Primary therapy outcome success (AUC = 0.5910, *P* = 0.01361).

In the meantime, the prognostic value of diverse clinicopathological parameters was also investigated. The K-M approaches disclosed that the tumor status (*cancer status* in Figure 6A and the *therapeutic efficiencies* in Figure 6B) and *new tumor event after initial treatment* (Figure 6C) could manifest the outcome between high- and low-risk groups. Moreover, ROC showed that new tumor event (AUC = 0.6233, *p* = 0.01992, Figure 7A) and primary therapy outcome success (AUC = 0.5910, *p* = 0.01361, Figure 7B) gained certain value to estimate patients’ survival, and the effect was weaker as compared to that from the PI. Several parameters were found to have some prognostic value with univariate analysis; however, only person neoplasm cancer status remained statistically significant with the confirmation by multivariate analysis (HR = 3.446, *p* = 0.029, Table 2).

**Table 2:** Univariate and multivariate Cox regression analysis of characteristics and prognosis index in LUSC

Characteristics	Patients	Univariate analysis		Multivariate analysis	
		HR (95% CI)	*p*-value	HR (95% CI)	*p*-value
Gender (male/female)	312/107	1.218 (0.847,1.753)	0.288		
Age (< = 65vs > 65)	258/155	0.8 (0.576,1.111)	0.183		
Dimension (> = median/< median)	151/142	0.762 (0.548,1.062)	0.108	1.563 (0.822,2.971)	0.173
Smoking (current smoking/no smoking now)	119/295	1.272 (0.888,1.821)	0.189	1.536 (0.832,2.834)	0.17
Distant metastasis (yes/no)	68/346	1.25 (0.843,1.852)	0.266	0.708 (0.258,1.943)	0.502
Lymph node metastasis (yes/no)	154/266	0.920 (0.670,1.263)	0.605	0.730 (0.329,1.615)	0.437
Tumor (T3–T4/T1–T2)	78/342	0.79 (0.534,1.171)	0.24	1.833 (0.757,4.438)	0.179
Pathologic stage (III–IV/I–II)	81/336	0.739 (0.503,1.086)	0.124	1.178 (0.425,3.262)	0.753
Location (central/peripheral)	124/78	0.716 (0.447,1.147)	0.165		
Radiation therapy (no/yes)	281/41	0.877 (0.530,1.451)	0.61		
Targeted molecular therapy (no/yes)	226/100	1.140 (0.755,1.720)	0.533		
EGFR mutation (no/yes)	237/16	1.03 (0.793,1.338)	0.822		
EML4-alk translocation (no/yes)	236/7	1.367 (0.552,3.385)	0.499		
Dlco predictive percent (< 80% /> = 80%)	83/35	1.732 (0.419,7.163)	0.448		
Karnofsky performance score (> = 70/70 <)	71/40	0.78 (0.425,1.432)	0.422		
Pre FEV1 (< 70%/ > = 70%)	62/63	1.318 (0.784,2.215)	0.298		
preFEV1 /FVC (< = 50%/50%–69% /> 70%)	83/45/13	1.249 (0.678,2.301)	0.475		
New tumor event after initial treatment (Yes/No)	223/85	2.031 (1.419,2.907)	< 0.001	0.739 (0.255,2.140)	0.577
Person neoplasm cancer status (with tumor/tumor free)	92/254	2.932 (2.055,4.184)	< 0.001	3.446 (1.139,10.426)	0.029
Primary therapy outcome success(SD + PD/CR + PR)	35/202	2.454 (1.473,4.088)	0.001	1.373 (0.617,3.053)	0.437
PI (High-risk/Low-risk)	215/214	2.068 (1.503,2.847)	< 0.001	1.928 (1.037,3.583)	0.038

### Functional evaluation of the lncRNAs by WGCNA

**Figure 8:**
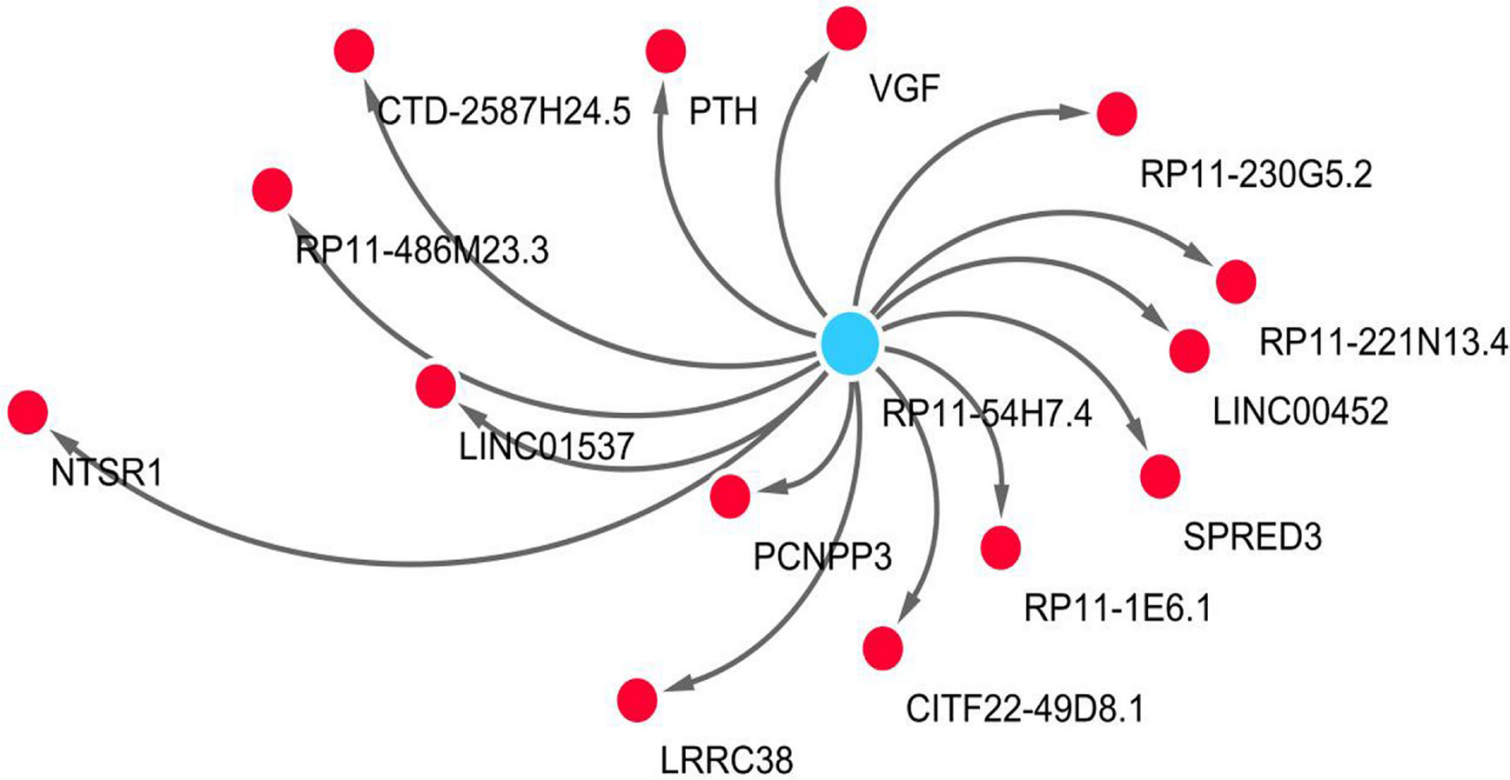
The network of lncRNA RP11-54H7.4 with co-expressed genes by WGCNA In the centric position, RP11-54H7.4 is the key lncRNA (blue node), surrounded with co-expressed genes (red nodes). The distance between the RP11-54H7.4 and the co-expressed genes is represented by co-expression weight. The network is conduct by Cytoscape 3.4 software.

The relevant genes of key lncRNA RP11-54H7.4 are RP11-486M23.3, PTH, RP11-1E6.1, LRRC38, NTSR1, CTD-2587H24.5, CITF22-49D8.1, PCNPP3, RP11-221N13.4, VGF, RP11-230G5.2, SPRED3 LINC01537 and RP11-221N13.4 (Table 3). The co-expression networks of lncRNA RP11-54H7.4 was visualized by WGCNA in the Figure 8.

**Table 3:** The co-expressed genes of key lncRNA RP11-54H7.4

key lncRNA	co-expressed gene	weight
RP11-54H7.4		
	LINC00452	0.661284074
	LINC01537	0.60362469
	RP11-486M23.3	0.616119829
	PTH	0.590041729
	RP11-1E6.1	0.655380384
	LRRC38	0.55758221
	NTSR1	0.525282159
	CTD-2587H24.5	0.542555868
	CITF22-49D8.1	0.592067422
	PCNPP3	0.658532282
	RP11-221N13.4	0.638936251
	VGF	0.628638631
	RP11-230G5.2	0.658032719
	SPRED3	0.571112779

### Validation of the expression of the five lncRNAs with GEO data

Finally, eight studies were screened out from GEO, including GSE19188, GSE30219, GSE33479, GSE37745, GSE50081, GSE73403, GSE74706 and GSE74777. However, only the expression of one key lncRNA, ZNF503-AS1, could be achieved in four datasets (GSE19188, GSE30219, GSE33479, GSE74706) provided ZNF503-AS1 expression data both in LUSC tissues and non-cancerous tissues, which showed the significantly lower level of ZNF503-AS1 in LUSC cases (Supplementary Table 1).

### Validation based on in-house clinical samples of LUSC

**Figure 9:**
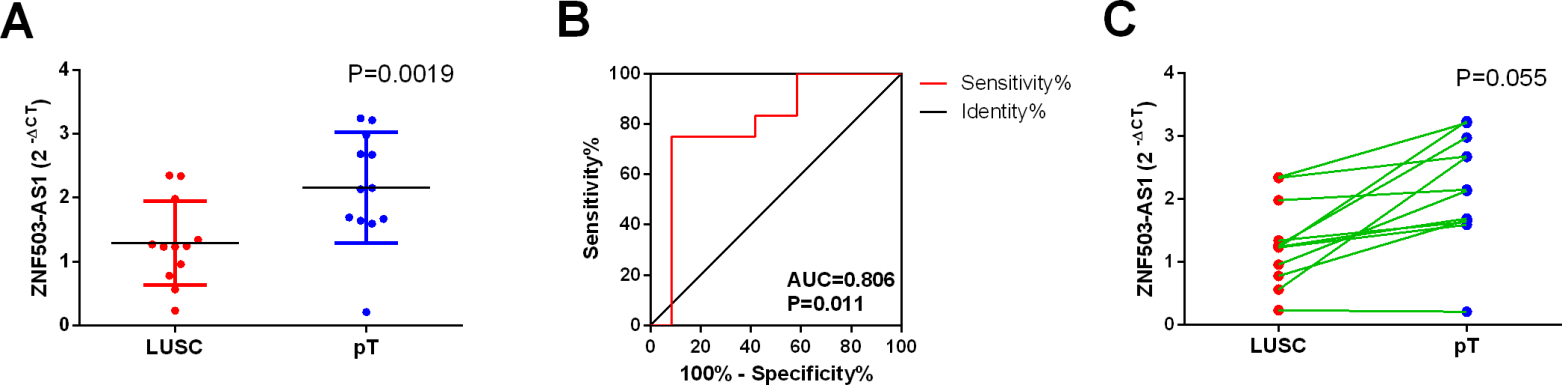
Validation of ZNF503-AS1 based on in-house clinical samples of LUSC (**A**) The expression of ZNF503-AS1 between para-tumorous lung tissues (pT) and LUSC (RT-qPCR); (**B**) ROC curve; (**C**) The correlation of ZNF503-AS1 between para-tumorous lung tissues (pT) and LUSC.pT: para-noncancerous tissues.

Since only ZNF503-AS1 was observed in GEO data, we performed real time RT-qPCR to confirm its expression in the clinical sample in house. The primers were listed as follows: Forward-5′-TGAGCGAGTTCGTACAGTGC-3′, Reverse-5′- TAGCATGTTAGCGCAGCCTT-3′. Among these small size of patients, 11 cases showed lower expression of ZNF503-AS1 in LUSC tissue than non-cancerous lung tissues and 1 cases showed higher expression. The mean expression level of ZNF503-AS1 was significantly lower than that of non-cancerous lung tissues (*P* = 0.0019, Figure 9A). Furthermore, the AUC of ZNF503-AS1 was 0.806 (*P* = 0.011, Figure 9B). However, due the small size of the patients, we could not assess the relationship between ZNF503-AS1 and the development of LUSC (*P* = 0.055, Figure 9C).

## DISCUSSION

LUSC is one of the most lethal malignancies worldwide. Several principal mechanisms have been documented over the past decades, including various molecule alterations, which could confer the oncogenesis and progression of LUSC [17, 41–45]. However, unlike LUAD, none specific biomarkers have been discovered to display the therapeutic efficiency and prognosis of LUSC. Therefore, the investigation of prognostic factors is of paramount importance for the management of LUSC patients.

Most recently, Liu et al. [46] assessed the prognostic value of lncRNA alteration frequencies via cBioPortal in lung cancers, including LUSC and LUAD. They found in LUSC, 624 lncRNAs had alteration rates > 1% and 64 > 10%. In LUAD, 625 lncRNAs had alteration rates > 1% and 36 > 10%. Among those, 620 lncRNAs had alteration frequencies > 1% in both LUSC and LUAD, while 22 were LUSC-specific and 23 were LUAD-specific. Twenty lncRNAs had alteration frequencies > 10% in both LUSC and LUAD, while 44 were LUSC-specific and 16 were LUAD specific. Since the genetic alteration includes gene amplification, mRNA upregulation, mRNA downregulation and all types of mutations, the clinical value of these genetic alterations of lncRNAs are somehow too generalized for clinical setting.

In the current study, we mined the public lncRNA data from TCGA and searched for a novel prognostic marker for LUSC. Interestingly, we found that the PI constructed from five differentially expressed lncRNAs could gain a good prognostic value for LUSC. As far as we know, this is the first investigation to generate a PI with RNA-seq based TCGA data for the survival evaluation of LUSC patients with a large cohort of 502 cases.

TCGA data with large-scale genomic analyses have rendered it possible to assess the molecular features related to LUSC outcome [47]. Huang et al. [48] explored 129 cases of the LUSC samples from TCGA with gene expression, microRNA expression, DNA methylation, and CNV data in 2015. They built the whole genome integrative network using variance inflation factor regression and isolated the lung cancer subnetwork with the Bayesian method. However, no lncRNA was involved. Gao et al. [49] also reported that 12 in 133 aberrant miRNAs were related to OS of TCGA LUSC cohort. Furthermore, a linear prognostic model containing seven miRNAs was constructed, which could predict patients’ survival with accuracy. These aforementioned studies provided a new and constructive study strategy of prognostic markers for LUSC. Nevertheless, no lncRNA was mentioned in the study.

By far, only two studies mined the lncRNA data from TCGA of LUSC. Wu et al. [50] investigated the differentially expressed genes (DEGs) based on the RNA-seq data of LUSC deposited in the TCGA database between LUSC samples and normal samples. Totally, they obtained 5162 DEGs, including seven upregulated lncRNAs (DIO3OS, HAR1A, FAM138B, BCYRN1, TERC, HCGA and PVT1). However, the relationship between these lncRNAs and survival was not established. Wei et al. [51] evaluated the altered lncRNA profiling in LUAD and LUSC with only the paired tissue samples of RNA sequencing or microarray data from TCGA and GEO. They observed that lncRNA expression pattern was distinct in LUAD and LUSC. Furthermore, a 6-lncRNA signature expression pattern (RP11-111M22.3, TOPORS-AS1, RP11-383C5.4, AC078883.3, AC007566.10 and AC011526.1) was observed to be notably related to the OS of LUSC. Meanwhile, another 6-lncRNA signature (CRNDE, CTA-292E10.6, CTD-3025N20.3, RP11-983P16.4, FLI1-AS1 and AC007879.5) was closely correlated to the PFS of LUSC. But this conclusion was based on the sequencing data of lncRNA with a small sample size of 16 paired LUSC and normal lung samples. We attempted to validate the prognostic value of these novel five-lncRNA signature with GEO data. Unfortunately, no sufficient survival data of these five lncRNAs was provided in theses GEO datasets, so the prognostic value of these five-lncRNA signature needs further validation with other methods in the future. This finding needs to be confirmed with a larger sample size.

In the current study, we first screened the differentially expressed lncRNAs, and next we selected those lncRNAs which tended to gain prognostic value which could be verified again by multivariate analysis. Finally, we constructed the PI with five novel lncRNAs: CYP4F26P, RP11-108M12.3, RP11-38M8.1, RP11-54H7.4 and ZNF503-AS1. And most importantly, this PI was proved to be independent prognostic indicator for LUSC.

We were also interested in the prospective molecular mechanisms of these five lncRNAs. Unfortunately, no publication was found of these lncRNAs and little was known on the functional mechanism of these five lncRNAs. To this end, we performed WGCNA to explore the related genes of the lncRNAs. Fourteen candidates were brought into the network, which could be the most correlative genes of these lncRNAs in LUSC.

Though we investigated the prognostic value of lncRNAs based on TCGA data in LUSC, other reports based on different techniques are also available. For instance, Zhou et al. [52] carried out an array-based transcriptional analysis of lncRNAs in 603 cases of NSCLC by repurposing microarray probes from three Gene Expression Omnibus (GEO) database (GSE37745, GSE31210 and GSE50081). They found that an expression pattern of eight lncRNAs was closely related to OS of NSCLC patients, including RP11-21L23.2, CTD-2358C21.4, RP11-94L15.2, GPR158-AS1, KCNK15-AS1, AC104134.2, RP11-701P16.5 and RP11-379F4.4. Multivariate regression and stratified analysis further suggested that the eight-lncRNA signature was independent clinical and pathological factors. Similarly, Tu et al. [53] mined lncRNA expression profiling in 739 lung cancer patients from GEO datasets. A risk score model was built up on the basis of the expression data of eight lncRNAs (AK021595, BC030759, AK000053, AK124307, BC020384, AK022024, CR615992 and AF085995) in the training dataset (GSE30219). They further validated the association between these lncRNAs and survival of lung cancer patients in another three independent testing sets (GSE31210, GSE37745 and GSE19188). However, both Zhou et al. [52] and Tu et al. [53] investigated the prognostic role of lncRNA in a general lung cancer on the whole, without separating LUAD from LUSC. Since LUAD and LUSC have absolutely distinct molecular mechanism, it is of great significance to perform the prognostic assessment separately. It is also unsurprising that the five lncRNAs in LUSC noted in the current study are not concordant with those reported from lung cancer [52, 53]. Therefore, the five lncRNAs may be candidate prognostic biomarkers for LUSC patients with substantial clinical implications.

To sum up, we assessed the RNA-Seq based lncRNA data with 502 cases of LUSC patients from TCGA and managed to construct a five-lncRNA signature, which could become a novel prognostic indicator for LUSC. However, the clinical role as well as the biological function of these five lncRNAs needs to be further verified with more experiments.

## MATERIALS AND METHODS

### TCGA dataset and analysis of the differentially expressed lncRNAs

RNA sequencing (RNA-Seq) data from individuals with LUSC, which were calculated on IlluminaHiSeq RNASeq platform, were achieved from TCGA data portal (https://cancergenome.nih.gov/.), containing 502 LUSC tissues and 49 adjacent non-tumorous lung tissue samples up to November 9, 2016. The expression data of lncRNAs were displayed as reads per million (RPM) and the expression level of each lncRNA was normalizated by Deseq package of R language for further analysis. The data were from TCGA, which is a community resource project providing available data for community research. Approval by a local ethics committee was not required because the current study adhered to the TCGA publication guidelines and data access policies. The RNA-Seq data of LUSC covered 60,483 mRNAs containing 7589 lncRNAs, which have been described by NCBI (https://www.ncbi.nlm.nih.gov/) or Ensembl (http://asia.ensembl.org/). Next the differentially expressed lncRNAs were calculated by EdgeR and DEseq (Padj < 0.05 and the absolute log2 FC > 1), respectively. The final selected lncRNAs were the integration between the two approaches. Student's *t* test (SPSS Inc., Chicago, IL, USA) was used for the statistical analyses of the differentially expressed level of these five lncRNAs between LUSC and non-cancerous lung tissues.

### Manufacture of prognostic signature with differentially expressed lncRNAs for LUSC

The differentially expressed IncRNAs of which the expression level was less than 1 in exceeded 10% of all subjects were removed from the prognostic analysis. In the meantime, clinicopathological features, including survival information, were also achieved from TCGA. Cases without sufficient clinical data were omitted., Finally, the prognostic analysis included a total of 478 samples with expression data from 4221 IncRNAs. The end-point of the LUSC patients in our study was set up with OS. The average follow-up period was 28 months for these LUSC patients being involved.

The prognostic value of lncRNAs, as well as the clinicopathological features, was firstly assessed by the univariate Cox proportional hazards regression (*p* < 0.05). The statistically significant indicators including lncRNAs and clinicopathological features were further confirmed with the multivariate cox regression model. An lncRNA based prognosis risk score was constructed on the basis of a linear combination of the expression level multiplied regression coefficient derived from the multivariate cox regression model (β) with the following formula as previously reported [54, 55].

Prognosis Index (PI) = exp_CYP4F26P_*β_CYP4F26P_+exp_RP11-108M12.3_*β_RP11-108M12.3_+exp_RP11-38M8.1_*β_RP11-38M8.1_+exp_RP11-54H7.4_*β_RP11-54H7.4_+exp _ZNF503-AS1_*β_ZNF503-AS1_.

Based on the cut-off of the median PI, LUSC patients were then divided into high-score and low-score groups [56]. Univariate and multivariate Cox proportional hazards regression analyses were further performed to investigate the prognostic effect of this prognosis risk score, and adjustments were made for tumor dimension, smoking status, distant metastasis, lymph node metastasis, tumor status, pathologic stage, new tumor event after initial treatment, person neoplasm cancer status, primary therapy outcome success and PI. Hazard ratio (HR) and 95% confidence intervals (CI) were calculated. The time-dependent receiver operating characteristic (ROC) curve analysis within 5 years as the defining point was also conducted with the R package “survivalROC”, to assess the predictive accuracy of prognostic model for time dependent disease outcomes. Kaplan-Meier survival curves were drawn to evaluate the relationship between all parameters (clinical inspects and prognosis risk score) and OS of LUSC patients [57].

ROC curve was applied to evaluate the predictive value of the risk score for patients’ outcome after first course of treatment. If two-sided *P value* was less than 0.05, statistical significance was considered. SPSS 22.0 software (SPSS Inc., Chicago, IL, USA) was selected for the statistical analyses.

### Functional evaluation of lncRNAs with WGCNA

Incorporated networks for the DELs are analyzed with WGCNA, which is capable of describing the correlation patterns gene expression profiles [58]. The WGCNA R package was used to evaluate the significance of the five DELs and their module membership by the ‘p.weighted’, which negatively indicates the correlation between the DELs and co-expressed genes in LUSC. In the first step, WGCNA was conducted by the threshold of p.weighted > 0.5 in our study. Next, the pairwise Pearson correlation was used to evaluate the weighted co-expression between all the probe set subjects in an adjacency matrix. The adjacency was defined by a ‘soft threshold’, which raises the absolute value of the co-expression by a ij=|cor(xi, xj)|β. In this study, the soft threshold was set at β = 3 with the scale-free topology criterion [59]. Following the identification of weighted correlation, features of the network were presented by Cytoscape 3.4.0.

### Analysis of the differentially expressed lncRNAs with GEO data

We also attempted to validate the findings from TCGA and the relevant mRNA-Seq datasets of LUSC from GEO was searched. The search strategy was as follows: (cancer OR carcinoma OR adenocarcinoma OR tumour OR tumor OR malignanc* OR neoplas*) AND (lung OR pulmonary OR respiratory OR respiration OR aspiration OR bronchi OR bronchioles OR alveoli OR pneumocytes OR “air way”). The expression level of each lncRNA was extracted for further analysis from all included data. Student's *t* test (SPSS 22.0 Inc., Chicago, IL, USA) was used for the statistical analyses of the differentially expressed level of these five lncRNAs between LUSC and non-cancerous lung tissues.

### Validation based on clinical samples of LUSC

To further verify the data from GEO, we performed real time RT-qPCR to detect the level of some lncRNAs with clinical LUSC samples (*n* = 12) from the First Affiliated Hospital of Guangxi Medical University as previously reported [60]. The Ethical Committee of First Affiliated Hospital of Guangxi Medical University, China approved the present study. All participating patients provided informed consent and agreement for the research use of the clinical samples. GAPDH was used as internal reference with the primers as follows: Forward-5′-TGAACGGGAAGCTCACTGG-3′, Reverse-5′-TCCACCACCCTGTTGCTGTA-3′. Paired-samples *t* test was performed to compare the difference of lncRNAs between LUSC and non-cancerous lung tissues with SPSS 22.0. We also drew Receiver operating characteristic (ROC) curves to assess the effect of lncRNAs to discriminate the LUSC from non-cancerous lung tissue.

## SUPPLEMENTARY MATERIALS TABLE



## Abbreviations

lncRNAs: long non-coding RNAs
LUSC: lung squamous carcinoma
OS: overall survival
TCGA: The Cancer Genome Atlas
DELs: differentially expressions lncRNAs
RNA Seq: RNA sequencing
HR: hazard ratio
ROC: receiver operating characteristic
AUC: area under the curve
CI: confidence interval
GO: Gene ontology
KEGG: Kyoto encyclopedia of genes and genomes
PPI: protein-protein interaction
CR: complete response
PR: partial response
SD: stable disease
PD: progressive disease.
