# Supplementary material for: Identification of a RNA-Seq based prognostic signature with five lncRNAs for lung squamous cell carcinoma
Source: Oncotarget. 2017 Apr 13;8(31):50761–73. doi: 10.18632/oncotarget.17098 (PMC5584202; doi:10.18632/oncotarget.17098)
Supplement: Supplementary file 1 [file oncotarget-08-50761-s001.pdf]

Identification of a RNA-Seq based prognostic signature with five lncRNAs for lung squamous cell carcinoma

Supplementary Materials

Supplementary Table 1: Validation of the expression of ZNF503-AS1 based on GEO datasets

Study	LUSC			Normal			*t*-test	
	n	Mean	SD	*n*	Mean	SD	*t*	*P*
GSE19188	27	−0.37698	0.281843	65	0.11456	0.334677	6.702	< 0.001
GSE30219	61	5.079881	0.450755	14	5.451717	0.471456	1.728	0.088
GSE33479	14	0.725709	0.748462	27	1.329858	1.042678	1.921	0.062
GSE37745	66	5.727597	0.616864					
GSE50081	43	4.310211	0.521205					
GSE73403	69	7.271944	0.392556					
GSE74706	8	−1.58171	1.840449	8	0.489941	0.505099	2.936	0.022
GSE74777	107	3.547633	0.183529					
